# Sustainable inverse-vulcanised sulfur polymers†

**DOI:** 10.1039/c8ra04446e

**Published:** 2018-08-06

**Authors:** Douglas J. Parker, Samantha T. Chong, Tom Hasell

**Affiliations:** Department of Chemistry, University of Liverpool Crown Street Liverpool L69 7ZD UK t.hasell@liverpool.ac.uk; Materials Innovation Factory, University of Liverpool Oxford Street Liverpool L69 7ZD UK

## Abstract

We demonstrate two renewable crosslinkers that can stabilise sustainable high sulfur content polymers, *via* inverse-vulcanisation. With increasing levels of sulfur produced as a waste byproduct from hydrodesulfurisation of crude oil and gas, the need to find a method to utilise this abundant feedstock is pressing. The resulting sulfur copolymers can be synthesised relatively quickly, using a one-pot solvent free method, producing polymeric materials that are shape-persistent solids at room temperature and compare well to other inverse vulcanised polymers. The physical properties of these high sulfur polymeric materials, coupled with the ability to produce them sustainably, allow broad potential utility.

## Abstract 2

We demonstrate two renewable crosslinkers that can stabilise sustainable high sulfur content polymers, *via* inverse-vulcanisation.

## Graphical abstract

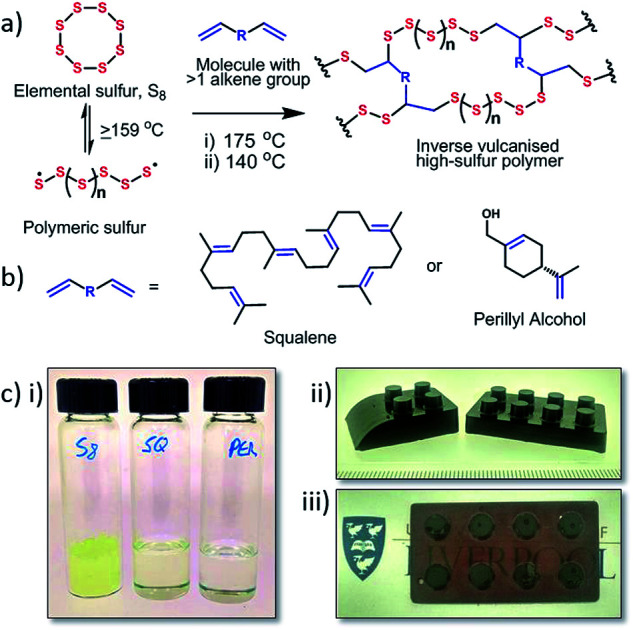

## Introduction

With the advent of the hydrodesulfurisation process to remove sulfur from natural gas and petroleum, sulfur has become a significant waste by-product with vast amounts of elemental sulfur being stockpiled at large refining sites as production outstrips demand.^1^ Although elemental sulfur has uses in specific areas of chemistry, for example, the production of sulfuric acid and fertilisers and in conventional vulcanisation, these processes make limited demands on the huge amount of available sulfur. This large abundance of sulfur makes it an economic feedstock for exploitation if suitable uses and reactions can be developed.

**Fig. 1:**
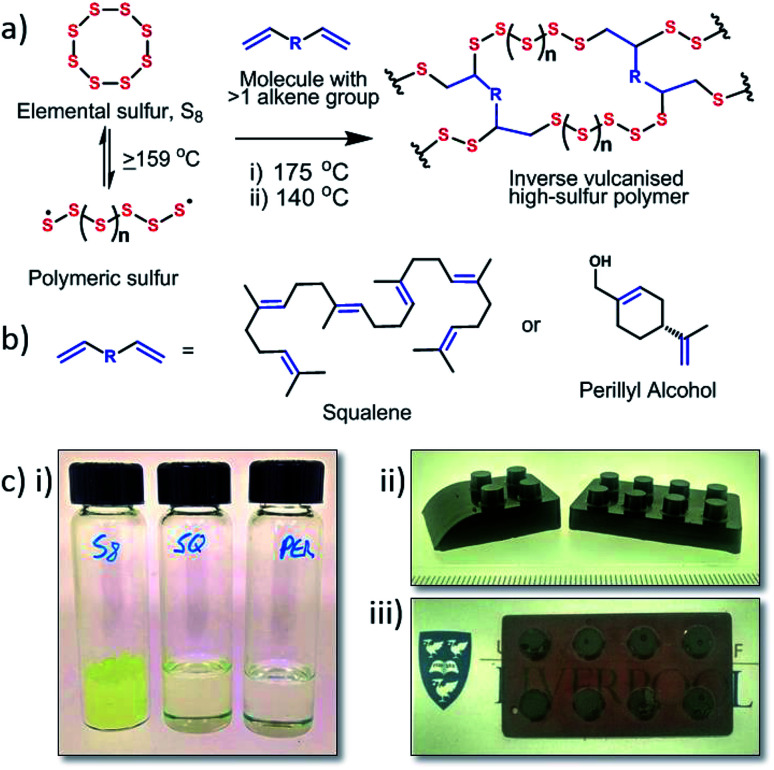
(a) General scheme outlining the synthesis of sustainable inverse vulcanised polymers (b) structures of the crosslinkers used, squalene (SQ) and perillyl alcohol (PER). (c) Photographs of (i) sulfur, squalene, and perillyl alcohol (L to R) and the resultant inverse-vulcanised polymers cast as pegged bricks: (ii) *S*-squalene copolymer, black solid, graduations show mm; (iii) *S*-perillyl alcohol copolymer, semi-transparent ruby red solid.

Under ambient conditions, elemental sulfur exists as a small cyclic molecule (S_8_) that on its own has poor physical properties. When sulfur is heated above its floor temperature (159 °C) it is able to undergo ring opening polymerisation. However, the resultant polymeric material is not stable and rapidly depolymerises back to elemental sulfur, due to the reversibility of S–S bonds.^2,3^ To prevent this depolymerisation, ‘inverse vulcanisation’ has been used to stabilise the polymeric material by crosslinking the sulfur with a small organic molecule, usually a diene, to create stable high sulfur content materials (Fig. 1). This discovery has generated much interest in sulfur polymeric materials synthesised *via* this inverse vulcanisation technique.^4^ First reported in 2013,^5^ this process, utilises a one-pot solvent free system and was a breakthrough for modern sulfur polymeric materials. The sulfur–diisopropenyl benzene (DIB) copolymer produced forms a solid material that is shape-persistent at room temperature, and has been demonstrated for multiple potential applications.^6,7^ However, DIB is a niche synthetic chemical relative to sulfur, and it would be preferable to couple the readily available waste sulfur with sustainable crosslinkers were possible. While crosslinker sustainability will impact less on ‘high-end’ applications of sulfur polymers such as LiS batteries,^5,8^ and optical devices,^6,9^ for applications with potential for wide distribution and use, such as heavy metal remediation^10,11^ or self-healing^12^ and antimicrobial materials,^13^ the sustainability and green credentials of the crosslinker may have more significance.

Recently there has been a surge of further reports of other inverse vulcanised polymers using a variety of crosslinkers.^4,14–19^ Although these new materials have made progress in improving the applications of sulfur materials, there are still issues to be addressed, such as molecular weight^14,15^ and cost. The cost can be attributed to either the crosslinker used^19^ or requiring multistep synthesis,^17,18^ when compared to simpler one-pot syntheses reported for commercially available crosslinkers.^20^ Bio-renewable crosslinkers make a particularly desirable target for crosslinking with sulfur, and prominent examples are limonene,^10^ vegetable oil,^21,22^ and di-allyl disulphide.^15^ Limonene is a by-product of the citrus industry, which isolates in excess of 70 000 tonnes per year from the zest of oranges,^23^ making it ideal to combine with waste sulfur. However, susceptibility to re-arrangement and hydrogen loss during the synthesis limits the molecular weight, and reduces the shape-persistency of the material. Vegetable oils similarly benefit from being abundant and readily sourced – and even used cooking oil can be employed,^22^ but these oils are only able to stabilise up to about 25–30 wt% of sulfur against depolymerisation to S_8_. Conversely, di-allyl disulfide, found in garlic oil, shows a remarkably high sulfur stabilisation capacity – up to 90 wt%.^15^

Exploring renewable crosslinkers for sulfur polymers, and improving the physical properties, will enable the development of polymeric sulfur materials for mass applications. Herein we report the synthesis of two sulfur copolymers from renewable crosslinkers – squalene and perillyl alcohol (Fig. 1b). These polymers are produced by a simple, green, highly atom efficient synthesis, and show favourable glass transition temperatures, sulfur stabilisation, and mercury uptake.

## Experimental

### Materials

The following compounds were used as received, without further purification; 1,3-diisopropenyl benzene (DIB, 97%, Sigma Aldrich), (*R*)-(+)-limonene (LIM, 97%, Sigma Aldrich), squalene (SQ, ≥98%, Sigma Aldrich) (*S*)-(−)-perillyl alcohol (PER, ≥95%, FG, Sigma Aldrich), sulfur (S_8_, sublimed powder, reagent grade, ≥99.5%, Brenntag UK & Ireland), mercury(ii) chloride (ACS, 99.5% MIN, Alfa Aesar UK) and methylmercury chloride (standard, 1000 μg mL^−1^, LGC Standards).

### Synthesis of crosslinked polymers

Synthesis of the sulfur copolymers was carried out in 100 mL round bottom flasks in aluminium heating blocks, with heating and stirring provided by electronic hotplates and magnetic stirrer bars. All reaction began by setting the hotplate to 175 °C, onto which a round bottom flask containing the required mass of sulfur was placed and allowed to fully melt. Upon fully melting, either squalene (SQ) or perillyl alcohol (PER) were added directly to the liquefied sulfur. The resulting mixture was stirred at *T* = 175 °C for five to twenty five minutes, (time dependent on the amount of crosslinker to react) by which time the reaction had changed to a thick dark brown liquid in the case of the SQ reactions and a ruby red solution for the PER reactions. At this point the reaction was transferred to a silicone mould and cured in an oven at 140 °C for 18 hours. Although the ratio of sulfur : crosslinker was varied in the experiments (50 : 50 to 90 : 10) the total mass of the reaction remained constant at 15.0 g. Full details of masses used and further information are reported in the ESI S1.†

### Characterisation

#### X-ray diffraction

In-house powder X-ray diffraction patterns (Fig. 3) were collected using a PANanalytical Empyrean powder diffractometer using CuKα radiation (Kα1 = 1.54060 Å, Kα2 = 1.54443 Å) and PIXcel3D detector. Samples were loaded into a space on the well-plate and run in transmission geometry. High-resolution synchrotron PXRD data were collected for samples held in 0.5 mm diameter borosilicate capillaries on the I11 beamline at Diamond Light Source (*λ* = 0.824 965 Å) using the Mythen-II positive sensitive detector in transmission geometry using a capillary spinner.

**Fig. 2:**
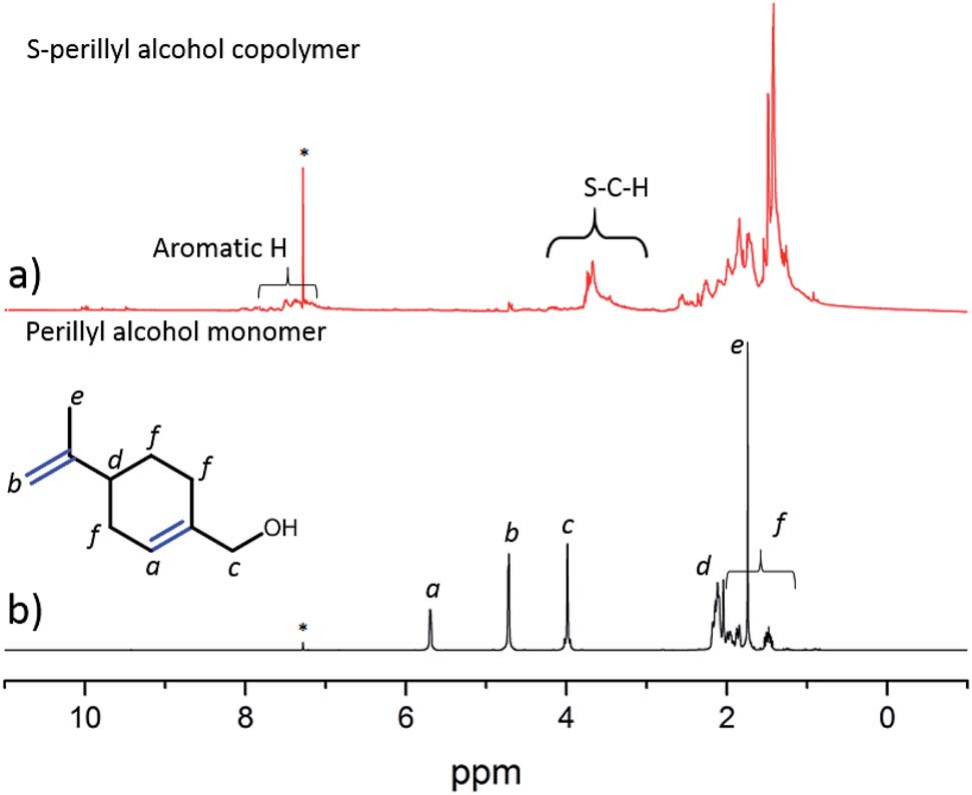
^1^H NMR for both the sulfur–perillyl alcohol 50 : 50 copolymer (a) and the perillyl alcohol monomer (b). Loss of vinylic proton resonances indicate a successful crosslinking by addition across the double bonds, though some aromatic H environments are detected, suggesting some possible hydrogen abstraction. The formation of new peaks in the 3.5–4 ppm region is consistent with the formation of C–S bonds. * = chloroform.

**Fig. 3:**
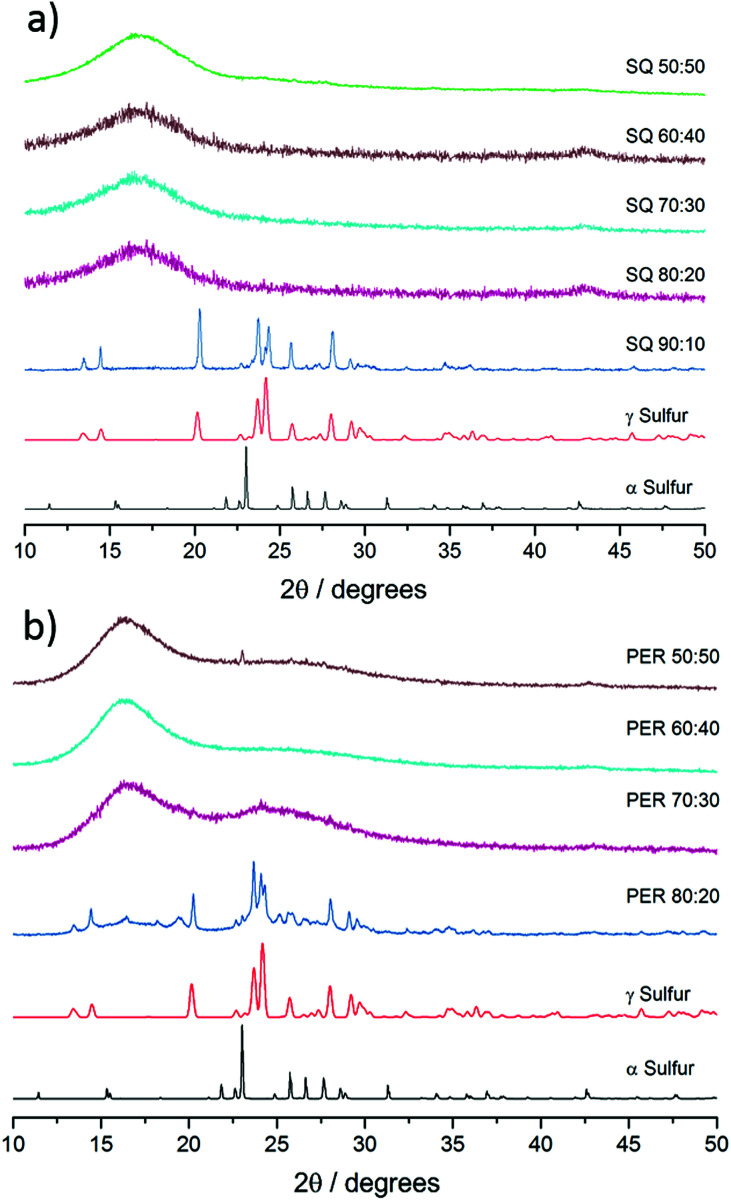
(a) Stacked p-XRD patterns for different sulfur : squalene copolymer ratios and polymorphs of elemental sulfur and (b) stacked p-XRD patterns for different sulfur : perillyl alcohol copolymer ratios and polymorphs of elemental sulfur.

#### Differential scanning calorimetry

Differential scanning calorimetry was performed using a TA Instruments Q200, with the DSC programmed as followed: equilibrate to 25 °C, then ramp to 150 °C at 5 °C per minute, then cool back to −80 °C and ramp to 150 °C.

#### Elemental analysis

Elemental analysis (CHNS) samples were submitted to the University of Liverpool, Chemistry Department Micro-Analysis service and run by Mrs Jean Ellis using an Elementar Vario Micro Cube.

#### Spectroscopic analysis (FT-IR and NMR)

Fourier Transformed Infrared spectroscopy (FT-IR) data was recorded on a Bruker TENSOR 27 FT-IR, between 400 cm^−1^ to 4000 cm^−1^ using an attenuated total reflectance accessory for 64 scans. Samples were analysed directly on the FT-IR without preparation. Nuclear Magnetic Resonance (NMR) samples were analysed using a Bruker Advance DRX (400 MHz) spectrometer. Proton (^1^H) NMRs were conducted at 96 scans and carbon (^13^C) NMRs were run for 1024 scans. All solution experiments were carried out at room temperature.

#### Inductively coupled plasma optical emission spectrometry

Inductively Coupled Plasma Optical Emission Spectrometry (ICP-OES) was performed on neat samples without digestion or further preparation, on an Agilent 5110 ICP-OES. Results for each sample were run at three different wavelengths and the average ppm recorded.

#### Gel permeation chromatography

Single detection Gel Permeation Chromatography (GPC) was performed using an Agilent 1260 Infinity II GPC/SEC system, two PLgel 5 μm MIXED-D columns and a PLgel 5 μm guard column, with samples detected by refractive index (RI). A mobile phase of chloroform was used with a flow-rate of 1 mL min^−1^ at 40 °C. GPC data was analysed using Agilent software and Agilent EasiCal PS-2 standards were used.

## Results and discussion

Squalene is a naturally occurring 30 – carbon terpene, found primarily in aquatic animals and some plants, and can now be produced synthetically from a yeast like fungus.^24^ Perillyl alcohol is a natural monocylic terpene found in many essential oils, it is a metabolite of limonene and is produced by plants *via* the mevalonate pathway. Perillyl alcohol can also be produced by use of a bioreactor.^25^ For copolymers of sulfur and one of these bio-renewable crosslinkers (perillyl alcohol or squalene), different ratios of sulfur to crosslinker were synthesised; 50 : 50, 60 : 40, 70 : 30, 80 : 20 (w/w%) for both crosslinkers and 90 : 10 w/w% for sulfur–squalene. These materials were then analysed by CHNS analysis to confirm that they contained the correct ratio of sulfur (see ESI, S2†). All copolymer compositions produced for both crosslinkers exhibited a glossy/glass like finish on the surface, with the squalene copolymers producing a hard black material and the perillyl alcohol copolymers producing dark ruby red translucent materials.

In testing both copolymers were insoluble in water, methanol and acetonitrile (no visible colour change, no detectable mass in the evaporated filtrate). However, perillyl alcohol copolymers were either fully or partially soluble in organic solvents such as chloroform and toluene (see ESI, S3†), whereas the squalene copolymers remained insoluble in all solvents. The low solubility of sulfur–squalene copolymers in organic solvents suggests that the large number of vinylic groups present in the crosslinker are available to react with the sulfur to form a dense crosslinked network. The insolubility of *S*-squalene polymers prevented NMR analysis. However, sulfur–perillyl alcohol copolymers were adequately soluble in deuterated chloroform to perform both ^1^H and HSQC NMR analysis (Fig. S4†). The resultant ^1^H NMR and comparison to the monomer (Fig. 2) shows the absence of vinylic peaks in the copolymer and a broadening of peaks between 1.25 and 2.5 ppm consistent with polymerisation. The appearance of peaks at ∼3.6 ppm is consistent with the formation of S–C–H positions by vulcanisation. The presence of small peaks in the ∼7–8 ppm range can most likely be attributed to some perillyl alcohol undergoing hydrogen abstraction from the cyclic system to form an aromatic derivative, as was found for the structurally related limonene.^10^ Hydrogen abstraction was also supported by higher than calculated C/H ratios observed by elemental analysis. Dehydrogenation of the cyclic system, thereby deactivating it to vulcanisation, would also lead to a more linear rather than crosslinked system, explaining the relatively high solubility.

FT-IR further confirmed reactions between sulfur and vinylic groups of the crosslinkers. When compared to the monomer there was an absence of C=C–H double bonds in both series of copolymers (see ESI, S5†). Both differential scanning calorimetry (DSC) and powder X-ray diffraction (pXRD) experiments were conducted to determine whether all the elemental sulfur had reacted and been incorporated homogenously throughout the material. Lack of crystallinity by XRD suggests the polymers are stable against depolymerisation – which would lead to the formation of S_8_ crystals within the polymer. The sulfur–squalene copolymers are stable against depolymerisation, as judged by pXRD, up to 80 wt% sulfur (Fig. 3a). By 90 wt% sulfur, crystalline peaks can be observed. The sulfur used as a feedstock in the synthesis is supplied as the α polymorph of sulfur, that being the lowest energy and most stable form at room temperature. On heating, it first transforms to the higher energy β-polymorph, before melting at 119 °C (see Fig. S6†). Interestingly, the crystalline sulfur that re-precipitated from the high sulfur content polymer did not revert to either the α or β form, but rather the meta-stable γ-polymorph. It is assumed this behaviour is caused by slow cooling of the un-stabilised sulfur trapped within the polymer. Perillyl alcohol stabilises up to 70 wt% elemental sulfur, before the copolymers start to show signs of depolymerisation, again to a γ polymorph of S_8_ crystals (Fig. 3b). In the case of the stable, amorphous polymeric forms, it can be noted that while both show a broad feature around 17°, the perillyl alcohol has a second feature at centred at approximately 25°, which we attribute to π–π stacking between aromatic groups formed through hydrogen abstraction.

**Fig. 4:**
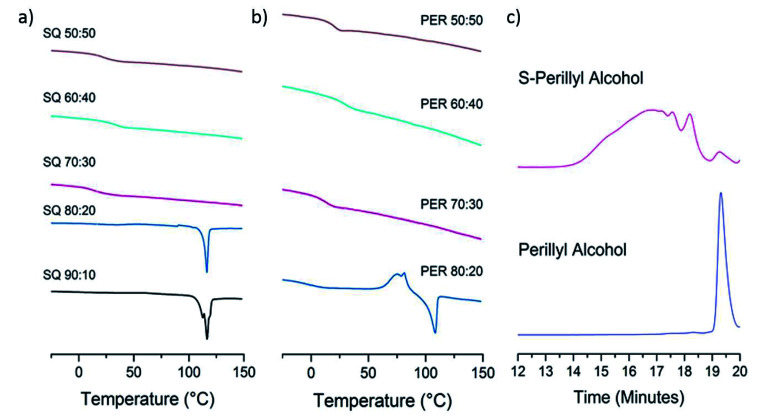
(a) Stacked DSC curves for different ratios of sulfur–squalene copolymers. The *T*_g_ of the polymers can be seen at 22 °C (50 : 50 wt% sulfur : squalene), 35 °C (50 : 50 wt% sulfur : squalene), and 14 °C (50 : 50 wt% sulfur : squalene). The 80 : 20 and 90 : 10 wt% sulfur : squalene products both show melting transitions for crystalline S_8_ at ∼120 °C. (b) Stacked DSC curves of different ratios of sulfur–perillyl alcohol copolymers. The *T*_g_ of the polymers can be seen at 20 °C (50 : 50 wt% sulfur : perillyl alcohol), 31 °C (50 : 50 wt% sulfur : perillyl alcohol), and 13 °C (50 : 50 wt% sulfur : perillyl alcohol). The 80 : 20 sulfur : perillyl alcohol product shows melting of crystalline S_8_ at ∼120 °C. (c) Stacked GPC comparison of perillyl alcohol monomer and sulfur copolymer.

**Fig. 5:**
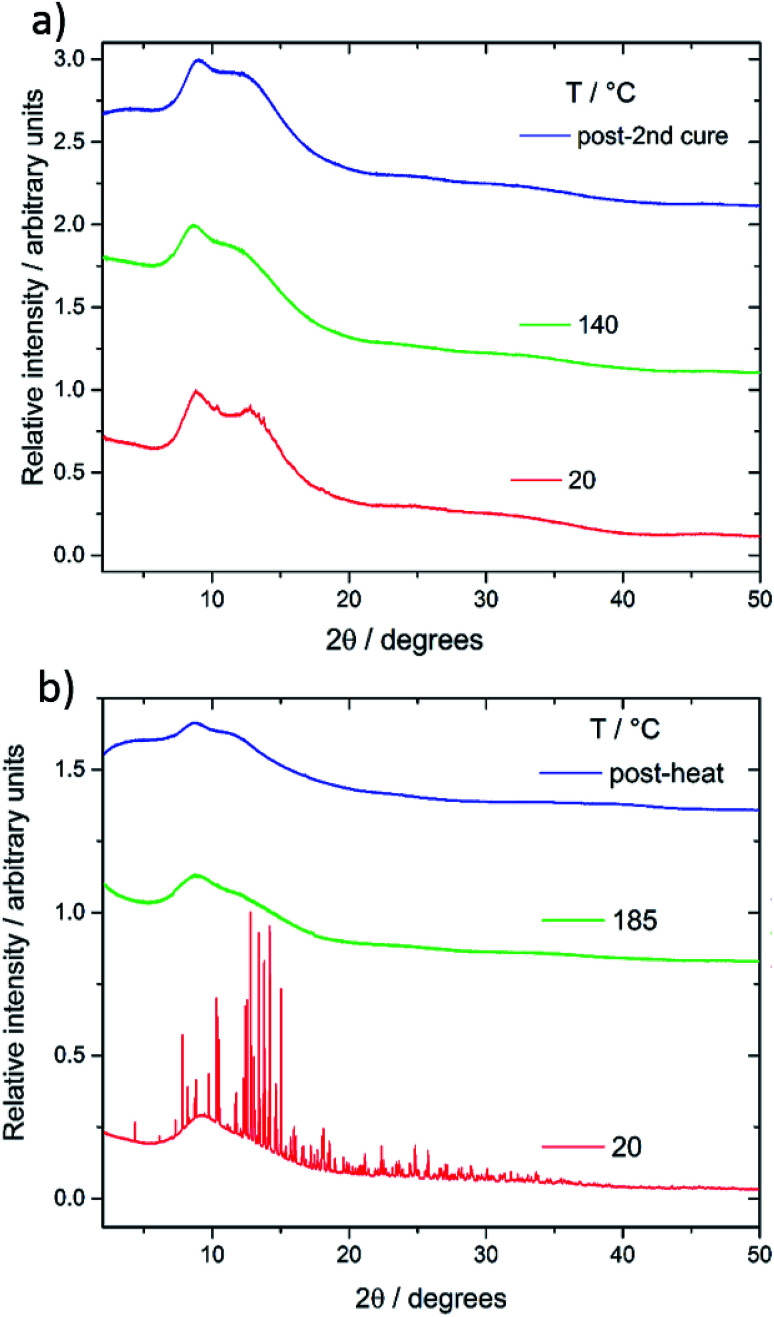
(a) Stacked pXRD patterns for 70 : 30 wt% sulfur–perillyl alcohol copolymer at room temperature, after heating to 140 °C to ‘cure’ the detected trace S_8_ crystals, and after 24 hours back at room temperature. (b) Stacked pXRD patterns for a slurry of sulfur and perillyl alcohol monomer, after heating to 185 °C to induce reaction, and after 24 hours at room temperature.

The lack of a crystalline melting transition by DSC (Fig. 4a and b) below 80 wt% sulfur for both copolymers, suggests the sulfur has been successfully reacted into a homogenous copolymer, whereas above these ratios the melting transition of S_8_ crystals can be detected. In terms of capacity to stabilise sulfur against depolymerisation, both copolymers perform comparably to other reported sulfur polymeric materials, of which most can stabilise only up to ∼80 wt% sulfur,^5,20^ and many only 60 wt%,^17^ 50 wt%^20^ and even ∼30 wt%.^22^ The detection of some S_8_ crystals by DSC in the case of 20 wt% squalene suggests DSC to be a more sensitive method of detecting the trace presence of S_8_ crystals than the pXRD results. Due to the concern that the laboratory pXRD was not detecting trace amounts of S_8_, that were picked up by DSC, it was decided to measure a sample at the extreme of sulfur content stabilisation by high intensity synchrotron pXRD. A 30 wt% perillyl alcohol, 70 wt% sulfur polymer was chosen, which showed broad and low intensity peaks (Fig. 5a). This indicates that the comparative accuracy of PXRD *vs.* DSC to detect trace S_8_ crystals is dependent on the source intensity, detection time, and sensitivity of the detector. The low intensity of the sulfur peaks suggests only an extremely small proportion of crystalline S_8_ is present (lower pattern, Fig. 5a). After heating above the melting point of sulfur (119 °C) and to our normal ‘cure’ temperature of 140 °C, the sample became completely amorphous (Fig. 5a, middle pattern). It was held at this temperature for a further hour before being cooled to room temperature, but no further crystallinity was observed even 24 hours later (Fig. 5a upper pattern). The change in crystallinity during direct synthesis was similarly assessed: an equal mass of sulfur and perillyl alcohol was heated till just over the melting point of sulfur, and stirred rapidly before being quickly cooled to room temperature. The intention of this was to ensure thorough mixing, without beginning the reaction. The resultant mixture, a fine yellow slurry, was packed into a 0.5 mm capillary and subject to variable temperature pXRD (Fig. 5b). The pattern of the loaded slurry (Fig. 5b lower pattern) shows a significant number of high intensity peaks, indicating the sulfur is still present as S_8_ crystals. These crystals are predominantly a phase mixture of the α and β forms, with the β form most prevalent, but with no γ form detected. The sample was then heated to 185 °C for one hour, losing all crystallinity (Fig. 5b, middle pattern). No crystallinity returned after 24 hours (Fig. 5b, upper pattern), suggesting polymerisation of the sulfur, rather than merely melting, occurred.

The solubility of the sulfur–perillyl alcohol products allowed gel permeation chromatography (GPC) to be performed (Fig. 4c) and this was performed on a 50 : 50 wt% ratio of sulfur to perillyl alcohol sample. When compared to sulfur–limonene copolymers made to the same ratio, and considering the structures of both crosslinkers are closely related, it is notable that the perillyl alcohol produces a higher molecular weight, and broader size distribution in the formed polymer. In comparison to polystyrene standards, the *S*-perillyl alcohol polymer would correspond to a *M*_w_ of 2261 and a *M*_n_ of 579, whereas *S*-limonene has been reported with an *M*_w_ of 242 and *M*_n_ of 210,^10^ or *M*_w_ of 904 and *M*_n_ of 493.^20^ Although these numbers should only be taken qualitatively due to the structural difference of these polymers to the standards, the higher molecular weight is presumably a contributory factor in the substantially higher glass transition temperature (*T*_g_) of *S*-perillyl alcohol in comparison to *S*-limonene (see below), and also in its greater degree of shape-persistence. However, stronger inter-molecular interactions resulting from the alcohol moiety may also influence these.

High-sulfur polymers have now been reported with a broad range of *T*_g_, and from soft rubbery solids to hard, brittle glasses. As such, there are no ‘better’ or ‘worse’ *T*_g_s, as the nature of polymer required will depend on the application – from compressible sponge like materials useful for oil–water separation,^26^ to hard inflexible materials for optical lenses.^27^ Instead, a broad range of *T*_g_s is preferable to allow diverse applications with appropriate choice of crosslinker for sulfur. However, so far it is only the industrially produced synthetic crosslinkers that have shown high glass transition temperatures, with most renewable crosslinkers leading to sub room temperature, or even sub 0 °C, glass transitions at equal weight ratios of sulfur to crosslinker, such as limonene (−21 °C),^10^ rapeseed oil (approx. −10 °C),^22^ diallyl disulfide (−14 °C to 4 °C)^15,28^ and myrcene (5–10 °C).^15^ When compared to these reported inverse-vulcanised polymers synthesised directly from renewable crosslinkers, both squalene and perillyl alcohol have comparatively high glass transition temperatures, at 21 and 20 °C respectively, for 50 wt% sulfur compositions (Fig. 4a and b). It has been previously observed that glass transition temperatures for inverse-vulcanised polymers tend to increase in proportion to the percentage of crosslinker added, such as for di-isopropenyl benzene (DIB),^5^ or dicyclopentadiene (DCPD).^20^ However, for both *S*-squalene and *S*-perillyl alcohol, the glass transition temperature, though increasing when going from 30 wt% crosslinker to 40 wt% crosslinker, seems to then reach a maximum, before dropping down to a lower temperature at 50 wt% crosslinker. The trend goes 14, 35, and 21 °C when going from 30, 40, and 50 wt% crosslinker for squalene, and similarly 20, 31, and 20 °C when going from 30, 40, and 50 wt% crosslinker for perillyl alcohol. It is possible that for both crosslinkers, a reasonably high proportion of sulfur is actually necessary for the polymerisation to proceed effectively. This may favour squalene radicals reacting with sulfur rather than undergoing intramolecular cyclisation, and for perillyl alcohol to react by addition across the double bonds, rather than through hydrogen abstraction. However, with glass transition temperatures over 30 °C possible for both of these high sulfur polymers, this puts them both in the glassy form at room temperature. As such they share more similarities with the inverse-vulcanised polymers reported from synthetic crosslinkers, such as *S*-DIB the most widely reported and applied inverse-vulcanised polymer (*T*_g_ 32 °C),^5^ and in complement to the previously reported sub room temperature *T*_g_ sulfur polymers from renewable crosslinkers.

### Re-processing

**Fig. 6:**
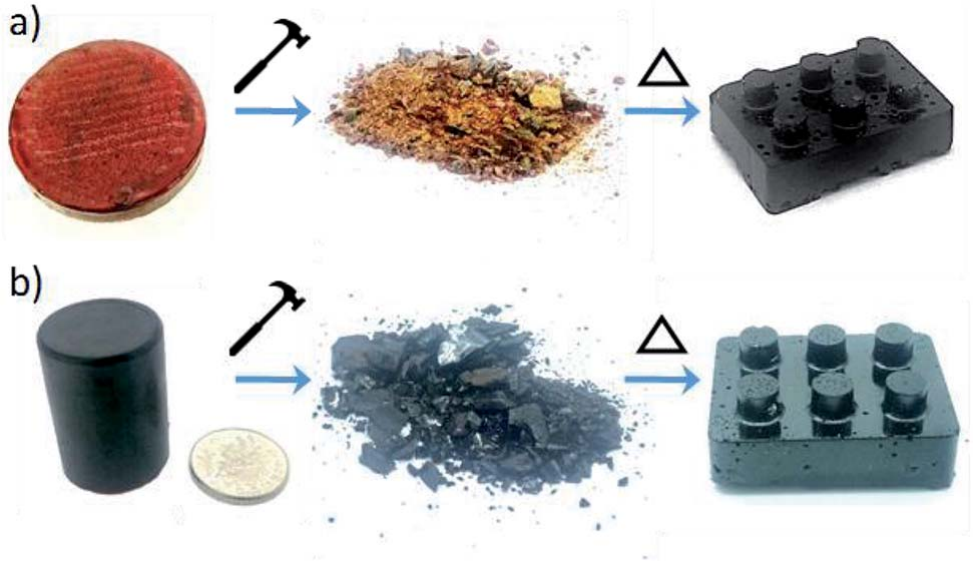
Sulfur polymer samples on, after breaking into powder, centre, and then after being reformed into a monolith again, right: (a) sulfur–perillyl alcohol copolymer, and (b) sulfur–squalene copolymer. Both samples were made with 50 wt% sulfur.

Linear polymers are normally thermoplastic and, by virtue of their solubility and melting transition, can often be re-processed into new solid forms, allowing recycling. Conversely, crosslinked organic polymers would be expected to be thermosets, and cannot normally be recycled. In recent years there has been increasing interest in a new class of crosslinked polymers becoming known as “Vitrimers”.^29,30^ These are crosslinked polymers with reversible bonds – strong organic glass formers that are able to change their topology through thermoactivated bond exchange reactions. At high temperatures, vitrimers can flow and behave like viscoelastic liquids, allowing them to be reprocessed like vitreous glass. Similarly, the reversibility of sulfur bonds in inverse vulcanised polymers has been shown to allow them to “heal” scratches,^6^ and even be fully re-processed.^12^ However, so far no inverse vulcanised polymers from renewable crosslinkers have been tested for this vitrimer behaviour, and we therefore tested both squalene and perillyl alcohol polymers. A block each of sulfur–squalene and sulfur–perillyl alcohol copolymers, both with 50 wt% sulfur, were smashed with a hammer and then placed back in moulds. These moulds were then placed in an oven at 155 °C and after 25 minutes the perillyl alcohol sample had liquefied to a thick red solution, at which point the mould was removed from the oven and allowed to cool. The sulfur–squalene took 40 minutes to melt into a thick black liquid, under slight compression. Once cooled both samples were removed from the moulds and were completely reformed copolymer blocks (Fig. 6).

It is perhaps unsurprising that the sulfur–perillyl alcohol co-polymer displays such thermoplastic behaviour, in view of its measureable molecular weight and solubility. However, that the fully crosslinked and insoluble sulfur–squalene copolymer can be processed in this way would not be expected if it were formed purely from irreversible carbon bonds.

### Heavy metal remediation

Mercury, and other heavy metals, are problematic for the environment as they are extremely toxic, persistent, and can bio-accumulate, leading to serious health issues such as heavy metal toxicity and even death.^31^ However, recent reports have shown inverse-vulcanised sulfur polymers can successfully remove inorganic mercury from aqueous solutions.^10,32^

Despite these reports, there has only been one study of an inverse-vulcanised sulfur copolymer and its ability to remediate organomercury compounds.^22^ Organomercury compounds are generally more toxic than their inorganic counterparts, being more readily absorbed by the body,^33^ and lipophilic nature.^34^ Methylmercury is one the major sources of mercury found in humans and was the cause of the Minamata Bay poisoning in the 1950s. Although anthropogenic sources of organomercury compounds in the environment have reduced greatly over the years, they can still be formed in the environment by the conversion of inorganic species.^35^ Therefore there is a need for sorbents that can efficiently remediate both organic and inorganic compounds.

**Fig. 7:**
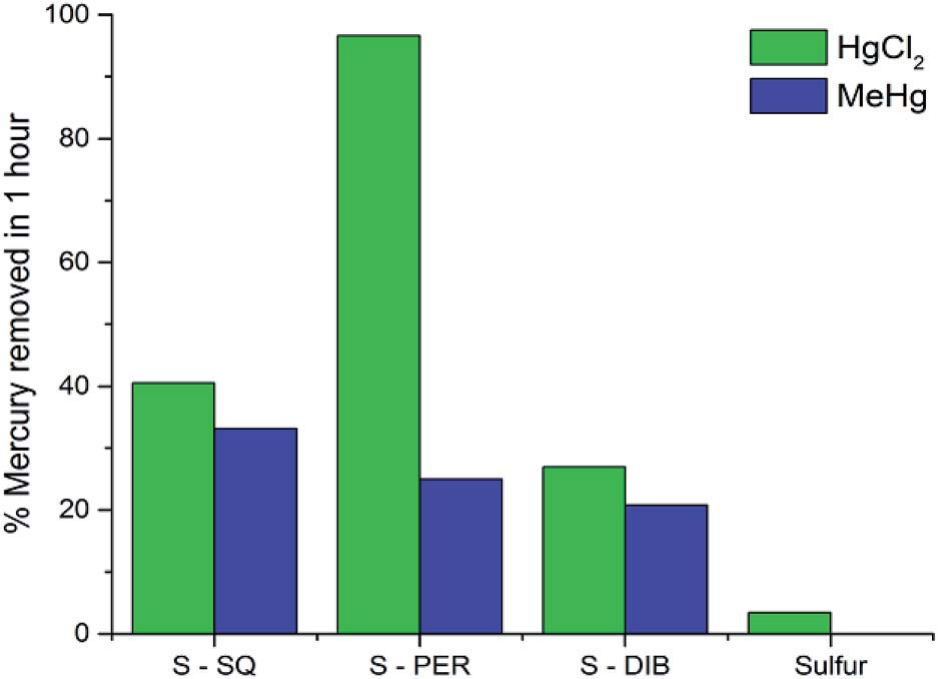
Mercury uptake results for mercury chloride and methylmercury chloride from a 2.5 ppm aqueous solution after 1 hour.

To determine how these copolymers compared to related materials, they were tested against sulfur–DIB and elemental sulfur for the adsorption of mercury from 2.5 ppm solutions (see ESI, S7†). All polymers tested depleted inorganic mercury from solution in an hour, with the perillyl alcohol copolymer removing in excess of 90% of HgCl_2_ in one hour and the sulfur–squalene copolymer showing an increased uptake of approximately 45% when compared to *S*-DIB. Also, both the perillyl alcohol and squalene copolymers show an increased affinity for organic mercury uptake compared to DIB, with squalene removing over 30% of the methyl mercury chloride present (Fig. 7). The 50% uptake increase when using *S*-SQ compared to *S*-DIB is likely attributed to the lipophilic nature of methylmercury chloride and the long carbon chain structure of the squalene crosslinker.

## Conclusions

Two renewable crosslinkers for inverse vulcanisation of elemental sulfur to form a stable polymer have been reported. The synthesis of each polymer is facile and compatible with the principles of green chemistry: solvent-free, high atom efficiency, and all feedstocks are either industrial waste (sulfur) or bio-renewable (crosslinkers), enabling significant potential for industrial scale up and use in bulk applications. The polymers reported are able to stabilise up to 70 wt% of sulfur against depolymerisation, have glass transitions above room temperature, and show vitrimer behaviour, allowing potential recycling. Both polymers demonstrated viability for mercury capture applications from aqueous streams.

## Conflicts of interest

There are no conflicts to declare.

## Supplementary Material

RA-008-C8RA04446E-s001
