# Correction: Sustainable inverse-vulcanised sulfur polymers

**DOI:** 10.1039/c8ra90071j

**Published:** 2018-08-29

**Authors:** Douglas J. Parker, Samantha Y. Chong, Tom Hasell

**Affiliations:** Department of Chemistry, University of Liverpool Crown Street Liverpool L69 7ZD UK t.hasell@liverpool.ac.uk; Materials Innovation Factory, University of Liverpool Oxford Street Liverpool L69 7ZD UK

## Abstract

Correction for ‘Sustainable inverse-vulcanised sulfur polymers’ by Douglas J. Parker *et al.*, *RSC Adv.*, 2018, **8**, 27892–27899.

The authors regret that the incorrect middle initial was given for Samantha Y. Chong in the original article. The corrected author list is as shown above.

The Royal Society of Chemistry apologises for these errors and any consequent inconvenience to authors and readers.

## Supplementary Material

