# Supplementary material for: Sustainable inverse-vulcanised sulfur polymers
Source: RSC Adv. 2018 Aug 6;8(49):27892–9. doi: 10.1039/c8ra04446e (PMC9083557; doi:10.1039/c8ra04446e)
Supplement: RA-008-C8RA04446E-s001 [file RA-008-C8RA04446E-s001.pdf]

Electronic Supplementary Material (ESI) for RSC Advances.
This journal is © The Royal Society of Chemistry 2018

Electronic Supplementary Information

Sustainable Inverse-Vulcanised Sulfur Polymers

Douglas J. Parker [1], Samantha Y. Chong [2] and Tom Hasell [1,*]

[1] Department of Chemistry, University of Liverpool, Crown Street, Liverpool, L69 7ZD, United Kingdom;
[2] Materials Innovation Factory, University of Liverpool, Oxford Street, Liverpool, L69 7ZD, United Kingdom;
* Correspondence: t.hasell@liverpool.ac.uk

S1. Synthesis details

The point to transfer the reaction mixture from the stirred vial to the mould was taken as the point at which an aliquot of the reaction mixture, when removed on a spatula and allowed to cool to room temperature, would no longer visibly separate to clear organic monomer, and precipitated yellow sulfur powder, but instead remain as a homogeneous brown/red viscous liquid.

50 wt% sulfur: The copolymerisation was carried out by following the general method written in the main paper with S_8 (7.50 g, 29.24 mmol) and either SQ (7.50 g, 18.26 mmol) or PER (7.50 g, 49.27 mmol).

60 wt% sulfur: The copolymerisation was carried out by following the general method written in the main paper with S_8 (9.00 g, 35.08 mmol) and either SQ (6.00 g, 14.61 mmol) or PER (6.00 g, 39.41 mmol).

70 wt% sulfur: The copolymerisation was carried out by following the general method written in the main paper with S_8 (10.5 g, 40.93 mmol) and either SQ (4.50 g, 10.96 mmol) or PER (4.50 g, 29.56 mmol).

80 wt% sulfur: The copolymerisation was carried out by following the general method written in the main paper with S_8 (12.0 g, 46.78 mmol) and either SQ (3.00 g, 7.30 mmol) or PER (3.00 g, 19.70 mmol).

90 wt% sulfur: The copolymerisation was carried out by following the general method written in the main paper with S_8 (13.5 g, 52.63 mmol) and SQ (1.50 g, 3.65 mmol).

Additionally a sample of sulfur-diisopropenyl benzene (DIB) was prepared using the same method, to be used in mercury uptake tests.

50 wt% sulfur:50 wt% DIB: The copolymerisation was carried out by following the general method written in the main paper with S_8 (7.50 g, 29.24 mmol) and DIB (7.50g, 47.40 mmol).

S2. Elemental Analysis

Sulfur-squalene copolymers

Sample	Calc. % Carbon	**Calc. % Hydrogen**	Calc. % Sulfur	**C : H ratio**	Actual % Carbon	**Actual % Hydrogen**	Actual % Sulfur	**C : H ratio**
**SQ 50:50**	43.87	**6.13**	50.00	**7.15 : 1**	43.57	**5.91**	50.52	**7.37 : 1**
**SQ 60:40**	35.09	**4.91**	60.00	**7.15 : 1**	33.92	**4.63**	61.35	**7.33 : 1**
**SQ 70:30**	26.32	**3.68**	70.00	**7.15 : 1**	20.68	**2.75**	76.47	**7.52 : 1**
**SQ 80:20**	17.55	**2.45**	80.00	**7.15 : 1**	13.20	**1.51**	84.93	**8.74 : 1**
**SQ 90:10**	8.77	**1.23**	90.00	**7.15 : 1**	8.38	**0.90**	91.18	**9.31 : 1**

Sulfur-perillyl alcohol copolymers

Sample	Calc. % Carbon	**Calc. % Hydrogen**	Calc. % Sulfur	**C : H ratio**	Actual % Carbon	**Actual % Hydrogen**	Actual % Sulfur	**C : H ratio**
**PER 50:50**	39.45	**5.30**	50.00	**7.44 : 1**	37.66	**4.73**	53.79	**7.96 : 1**
**PER 60:40**	31.56	**4.24**	60.00	**7.44 : 1**	32.25	**3.97**	60.51	**8.12 : 1**
**PER 70:30**	23.67	**3.18**	70.00	**7.44 : 1**	24.89	**2.98**	69.75	**8.35 : 1**
**PER 80:20**	15.78	**2.19**	80.00	**7.44 : 1**	15.88	**1.85**	80.04	**8.58 : 1**

S3. Solubility tests for sulfur-perillyl alcohol

Determined by placing 100 mg of solid in 10 mL solvent and agitating overnight. Samples that have fully dissolved after 24 hours have been given a solubility of ≥ 10.0 mg/mL.

**Solvent**	Solubility (mg/mL)	**Solvent**	Solubility (mg/ml)
**Acetone**	0.15	**Methanol**	Insoluble
**Acetonitrile**	Insoluble	**THF**	≥ 10.0
**Chloroform**	≥ 10.0	**Toluene**	≥ 10.0
**Hexane**	0.31	**Water**	Insoluble

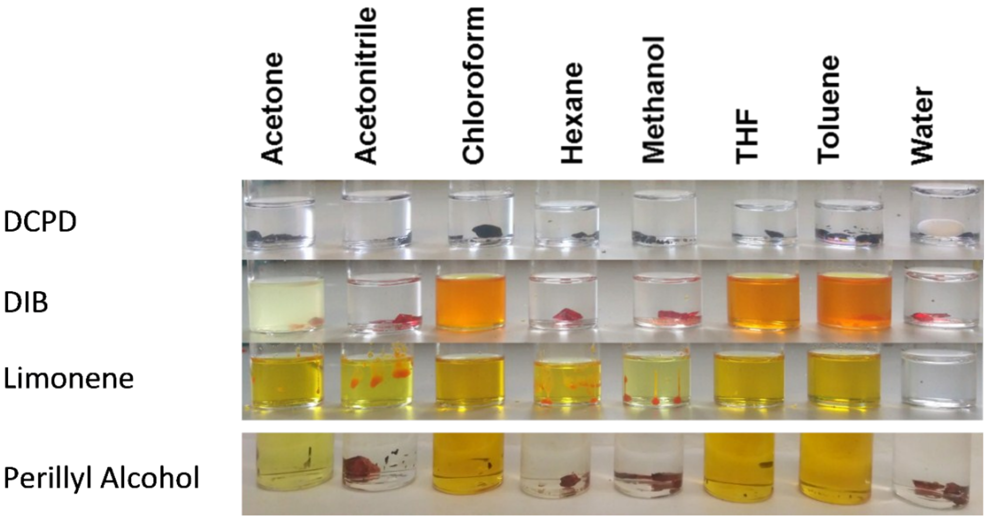

Figure S3. Photographs of sulfur polymers in a range of solvents. S-perillyl alcohol shows higher solubility than S-DCPD (made from dicyclopentadiene)[1], lower solubility than S-limonene [2], and is comparable to S-DIB [3].

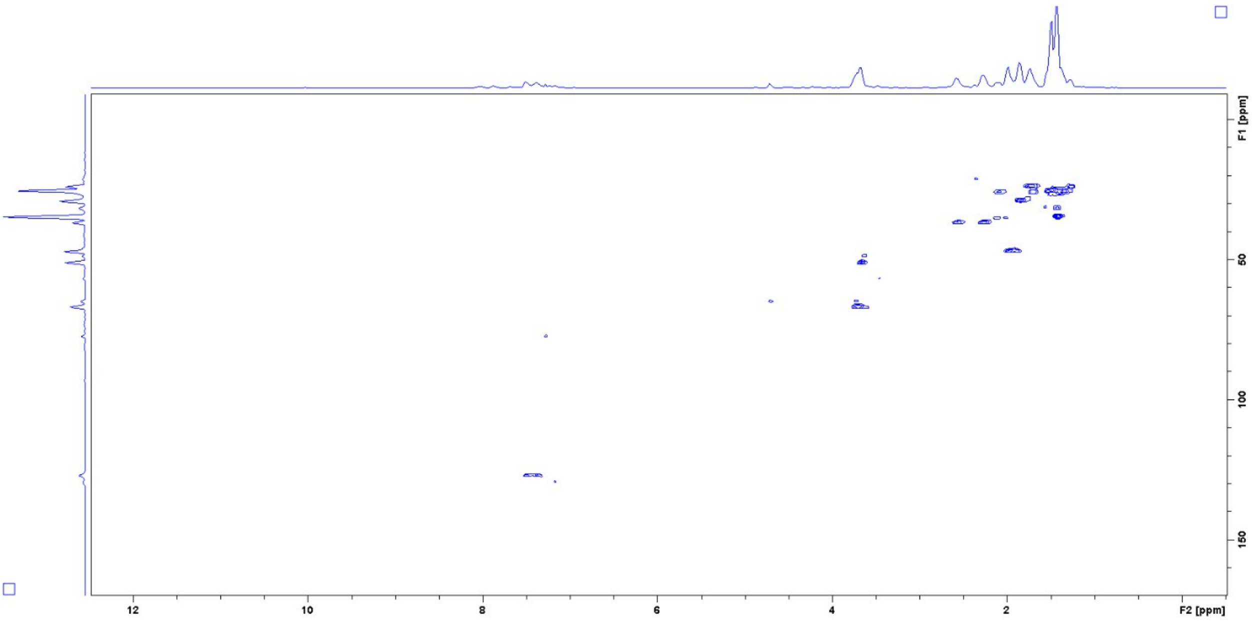

Figure S4: HSQC NMR of sulfur-perillyl alcohol dissolved in chloroform. It can be noted that the proton positions at ~7.3-7.6 ppm correspond to a shift of ~125 ppm, corresponding to an aromatic environment.

S5. FT-IR

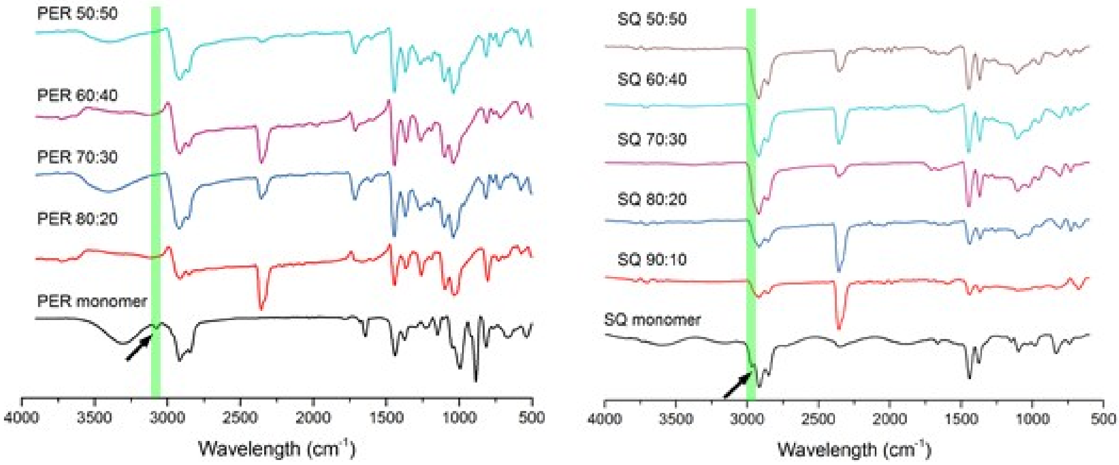

Figure S5. FT-IR spectra for both crosslinkers at different ratios. In both spectra the C=CH bond region is highlighted and the peak is annotated with an arrow in the monomers

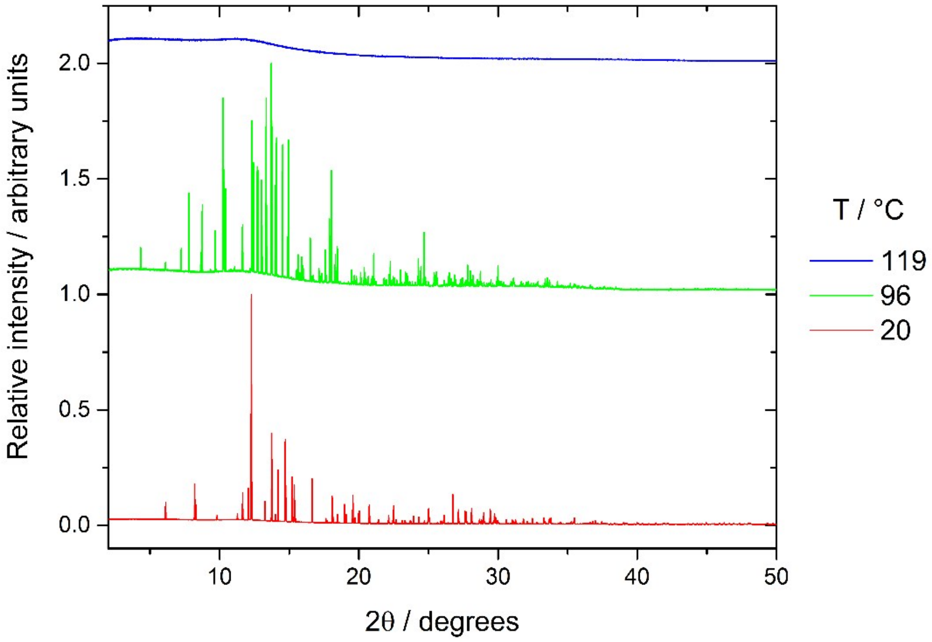

Figure S6. Variable temperature PXRD patterns (λ = 0.824965 Å) of pure sulfur. A capillary of the as-supplied sulfur feedstock was heated while collecting diffraction data. At room temperature the sulfur exists as the stable α-polymorph. At 96 °C, the sulfur underwent a phase transition to the β-polymorph, before finally melting at 119 °C.

S7. Mercury Uptake Testing

Inorganic mercury tests

A stock solution of mercury was made by dissolving $HgCl_2$ (338 mg) in deionised water (250 mL) to produce a concentration of 1000 ppm of $HgCL_2$. This was then used to prepare the test solutions of 2.5, 5, 10, 20 and 40 ppm by serial dilutions. Samples to be tested were coarsely ground and screened through a 45 mesh sieve before being accurately weighed (600 mg) into 15 mL centrifuge tubes to which 12 mL of each $HgCl_2$ solution was added, via single channel pipette. Tubes were capped and placed on a tube roller for 1 hour with medium agitation. After 1 hour, the tubes were removed and the solutions filtered into clean sample vials, using a 0.45 µm filter and a polypropylene syringe. Samples were analysed via ICP-OES using an Agilent 5110 spectrometer. Uptake values were calculated by difference, when compared to a control samples with no sorbent present and prepared at the same time.

Organic mercury tests

A 2.5 ppm solution of methylmercury chloride (MeHgCl) was prepared from a 1000 ppm stock solution (LGC Standards) and deionised water. Samples to be tested were coarsely ground and screened through a 45 mesh sieve before being accurately weighed (600 mg) into 15 mL centrifuge tubes to which 12 mL of the MeHg solution was added, via single channel pipette. Samples were capped and placed on a tube roller for 1 hour with medium agitation. After 1 hour, the tubes were removed and the solutions filtered into clean sample vials, using a 0.45 µm filter and a polypropylene syringe. Samples were analysed via ICP-OES using an Agilent 5110 spectrometer. Uptake values were calculated by difference, when compared to a control sample with no sorbent present and prepared at the same time.

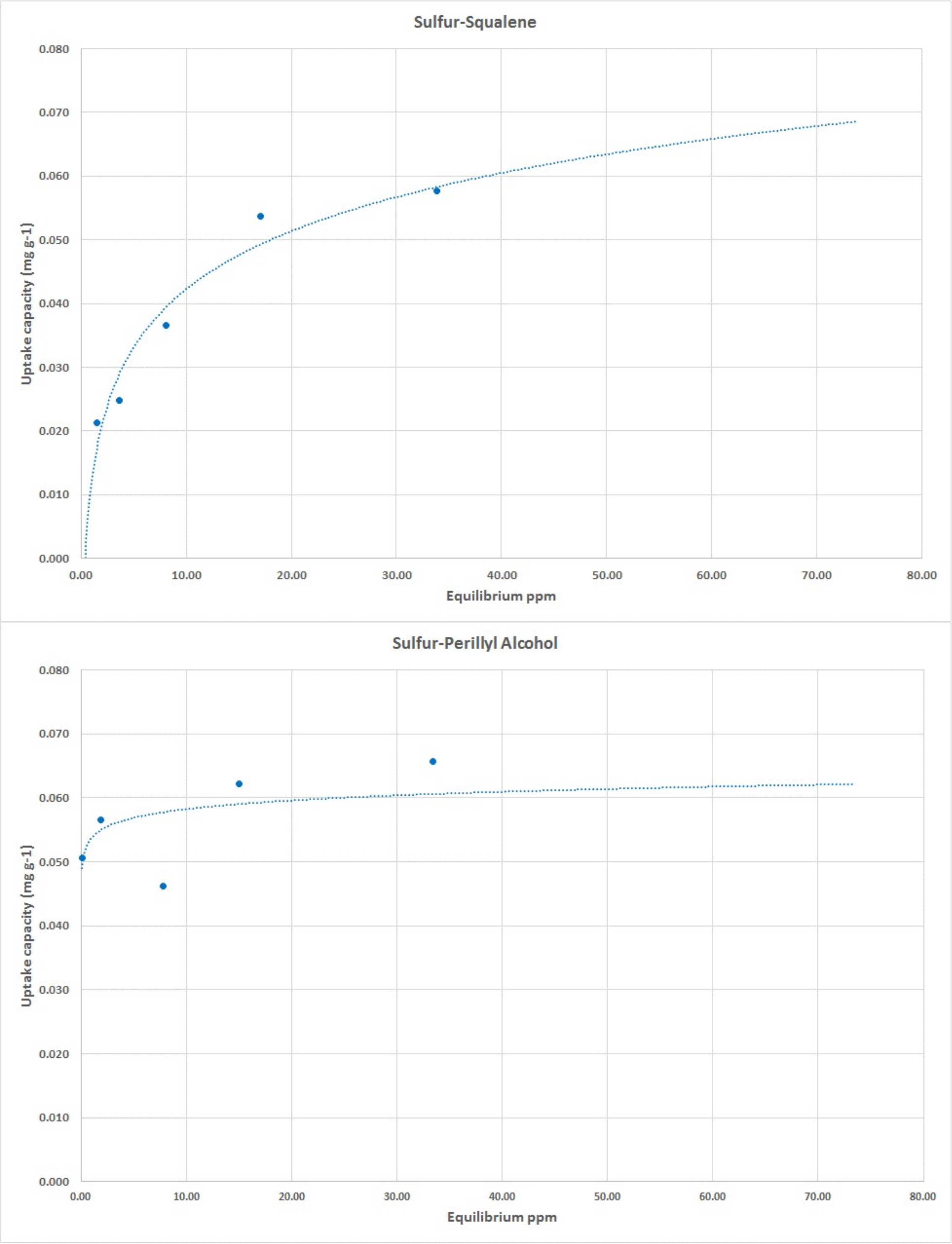

Figure S8. Uptake capacities for both 50:50 sulfur-squalene (top) and sulfur-perillyl alcohol (bottom) copolymers. Results show the uptake of mercury chloride from an aqueous solution using the method described above.

References:

1. Parker, D.J.; Jones, H.A.; Petcher, S.; Cervini, L.; Griffin, J.; Akhtar, R.; Hasell, T. Low cost and renewable sulfur-polymers by inverse vulcanisation. *Journal of Materials Chemistry A* **2016**.
2. Crockett, M.P.; Evans, A.M.; Worthington, M.J.H.; Albuquerque, I.S.; Slattery, A.D.; Gibson, C.T.; Campbell, J.A.; Lewis, D.A.; Bernardes, G.J.L.; Chalker, J.M. Sulfur-limonene polysulfide: A material synthesized entirely from industrial by-products and its use in removing toxic metals from water and soil. *Angewandte Chemie International Edition* **2015**, *55*, 1714-1718.
3. Chung, W.J.; Griebel, J.J.; Kim, E.T.; Yoon, H.; Simmonds, A.G.; Ji, H.J.; Dirlam, P.T.; Glass, R.S.; Wie, J.J.; Nguyen, N.A.*, et al.* The use of elemental sulfur as an alternative feedstock for polymeric materials. *Nat Chem* **2013**, *5*, 518-524.
